# Reliability of biochemical assays of nitric oxide biomarkers in human skeletal muscle tissue samples

**DOI:** 10.1371/journal.pone.0357952

**Published:** 2026-09-11

**Authors:** Raghini Rajaram, Matthew I. Black, Chenguang Wei, Andrew M. Jones, Paul G. Winyard, Anni Vanhatalo

**Affiliations:** University of Exeter Medical School, Faculty of Health and Life Sciences, University of Exeter, St Luke’s Campus, Exeter, United Kingdom; Dynamical Business & Science Society - DBSS International SAS, COLOMBIA

## Abstract

Accurate measurements of nitrate and nitrite concentrations in human skeletal muscle are fundamental to further our understanding of their role in nitric oxide (NO) homeostasis. We investigated the effects of saline wash and immediate processing with a potassium ferricyanide-containing nitrite-preserving stop solution (to remove the effects of haemoglobin from blood surrounding the tissue) on human skeletal muscle nitrate and nitrite concentrations. Skeletal muscle tissue was collected from 24 healthy adults and processed with and without saline wash, and with and without stop solution. Tissue samples (n = 17) washed in saline had significantly lower nitrate concentration (p < 0.01) and lower inter-sample variability (45 ± 23 nmol/g) compared to tissue samples not washed in saline (104 ± 52 nmol/g), with no correlation and high bias (−59 ± 49 nmol/g) between the two methods. Nitrite concentration was not significantly different between tissue samples washed in saline (1.94 ± 0.90 nmol/g) and samples not washed in saline (1.55 ± 0.86 nmol/g), with no correlation and minimal bias (0.39 ± 0.85 nmol/g) between the two methods. Addition of nitrite-preserving stop solution did not result in significant differences in concentrations of nitrate or nitrite. In summary, although our study is limited by the lack of Hb and Mb measurements, saline washing of biopsied tissue samples improved the reliability of nitrate and nitrite concentrations measured in human skeletal muscle.

## Introduction

The importance of the gaseous signalling molecule, nitric oxide (NO), in various mammalian physiological functions such as blood pressure regulation, metabolism, skeletal muscle contractility, immunity, cognitive function, and neurotransmission is well established [1–6]. NO is produced in mammalian tissues from an oxygen-dependent five electron oxidation of the amino acid, l-arginine, in a reaction catalysed by the nitric oxide synthase (NOS) enzymes [7]. Due to its short half-life in mammalian tissues, NO is rapidly oxidised to the more stable metabolites, nitrate (NO_3_^−^) and nitrite (NO_2_^−^). NO is also produced by the reduction of NO_3_^−^ and NO_2_^−^, which may be especially important in older age, clinical conditions (e.g., hypertension), and in conditions of hypoxia and acidosis when the function of the NOS pathway may be impaired [4]. NO homeostasis is maintained in the body through a negative feedback loop between the canonical NOS pathway and the NO_3_^−^ - NO_2_^−^ - NO pathway [8].

The presence of neuronal NOS (nNOS) and the NO oxidising haemoprotein, oxymyoglobin, suggests that skeletal muscle could play an important role in NO metabolism [9]. The observation of a NO_3_^−^ gradient from skeletal muscle to blood in rodents [9,10] and in some [11–13] but not all [14] human studies has led to suggestions that skeletal muscle could act as a ‘NO_3_^−^ reservoir’ to be utilised in times of reduced NO bioavailability [15]. The expression of sialin, a NO_3_^−^ transporter, and xanthine oxidoreductase (XOR), which has NO_3_^−^ and NO_2_^−^ reductase activity [9,10,16,17], have also been confirmed in human skeletal muscle [12] supporting the hypothesis that human skeletal muscle has the necessary apparatus for the transport, storage, and metabolism of NO_3_^−^ [5,18]. Given the large skeletal muscle mass in the human body [19], the capability of skeletal muscle for NO_3_^−^ storage and reduction to NO through XOR would be advantageous for regulation of whole body NO homeostasis. To better understand the role of human skeletal muscle in NO homeostasis and develop interventions to counter age-related decline in cardiovascular health and functional capacity from reduced NO bioavailability, it is important to ensure reliable measurements of NO_3_^−^ and NO_2_^−^ concentrations in human skeletal muscle tissue samples.

NO_2_^−^ is rapidly lost in biological samples as demonstrated by the 11–13 min half-life in blood [20]. Haemoproteins such as haemoglobin (Hb) in blood and myoglobin (Mb) in muscle contain the ferrous (Fe^2+^) haem group. The reaction of NO_2_^−^ with Fe^2+^ haem in oxyhaemoglobin (oxyHb) and oxymyoglobin (oxyMb) leads to oxidation of NO_2_^−^ to NO_3_^−^, while the Fe^2+^ haem in deoxyhaemoglobin (deoxyHb) and deoxymyoglobin (deoxyMb) is involved in the conversion of NO_2_^−^ to nitrosylHb (HbNO) [21]. In this process, the ferrous haem is converted to ferric (Fe^3+^) haem in methaemoglobin (metHb) and metmyoglobin (metMb). The above reactions are summarised below for oxyHb and deoxyHb:

oxyHb + NO_2_^−^ → metHb + NO_3_^−^
deoxyHb + NO_2_^−^ → metHb + NO
deoxyHb + NO → HbNO
oxyHb + NO → metHb + NO_3_^−^

NO_2_^−^−preserving stop solution (“stop solution”) was originally added to blood samples to improve the reliability of NO_3_^−^ and NO_2_^−^ concentration ([NO_3_^−^] and [NO_2_^−^]) measurements in whole blood and red blood cells due to the effect of Hb noted above [22]. Potassium ferricyanide (K_3_Fe(CN)_6_) in the stop solution converts the Fe^2+^ haem in Hb and Mb to the Fe^3+^ haem in metHb and metMb and hence prevents the oxidation of NO_2_^−^ to NO_3_^−^. A diluted (1:9 v/v) form of this stop solution has been used when rodent skeletal muscle samples were processed for [NO_3_^−^] and [NO_2_^−^] measurements as the concentration of haemoproteins in skeletal muscle was considered to be lower than in blood [23].

[NO_3_^−^] and [NO_2_^−^] in human skeletal muscle samples have been measured in a limited number of studies with variable results both at baseline and post−NO_3_^−^ supplementation [11–14,24–27]. While this could be due to differences in age, sex, diet, physical activity, and health status of the participants studied, the processing of samples and the techniques used for the measurement of [NO_3_^−^] and [NO_2_^−^] in skeletal muscle samples is still evolving and could also contribute to the variability. The skeletal muscle homogenisation procedures for [NO_3_^−^] and [NO_2_^−^] measurements have been developed by Park *et al.*, 2021 [23] using rodent skeletal muscle. One of the key differences in the processing of rodent and human skeletal muscle samples is that the rodent muscle is extracted after perfusion of the organs with saline [9,10,15,23], while the biopsied human skeletal muscle is blotted with sterile gauze to remove excess blood [12–14,24–27]. It is therefore possible that human skeletal muscle tissue contains more Hb from blood than saline-perfused rodent tissue, thereby affecting the measured concentrations of NO_3_^−^ and NO_2_^−^ in human skeletal muscle samples.

The time at which the tissue samples are homogenised with the stop solution could also potentially influence [NO_3_^−^] and [NO_2_^−^] measurements as cell lysis will enable effective preservation of [NO_3_^−^] and [NO_2_^−^] through the action of K_3_Fe(CN)_6_ on Hb and Mb in all cellular components of the muscle fibres. In rodent studies, [NO_3_^−^] and [NO_2_^−^] varied between different leg muscles, and [NO_3_^−^] levels were marginally higher in smaller compared to larger tissue samples [23]. Human muscle samples for [NO_3_^−^] and [NO_2_^−^] measurements are typically collected from *m. vastus lateralis* but the potential variability in [NO_3_^−^] and [NO_2_^−^] in different tissue samples collected from the same muscle is unknown. Since the amount of skeletal muscle tissue obtained from each biopsy is variable in humans (i.e., ~ 20–150 mg), it is also important to establish the possible effect of sample weight on [NO_3_^−^] and [NO_2_^−^] in human skeletal muscle samples.

The primary aim of this study was to analyse the combined procedural effects of saline wash and immediate homogenisation of the biopsied tissue with stop solution on human skeletal muscle [NO_3_^−^] and [NO_2_^−^] measurements. We hypothesised that washing the human skeletal muscle tissue with saline before blotting with sterile gauze, in combination with the addition of the stop solution and homogenisation immediately upon sample collection, would remove more extracellular Hb compared to only blotting with gauze, and facilitate the immediate action of K_3_Fe(CN)_6_ on any remaining extracellular Hb and intracellular Mb. The removal of Hb and formation of metHb and metMb by K_3_Fe(CN)_6_ would preserve NO_2_^−^ in the tissue samples by preventing the oxidation of NO_2_^−^ to NO_3_^−^, and hence the [NO_2_^−^] in samples washed with saline would be higher than in the samples only blotted with gauze. The removal of Hb and formation of metHb and metMb could also result in lower [NO_3_^−^] in samples washed with saline compared to the samples only blotted with gauze, albeit the small change in [NO_3_^−^] could be difficult to detect because of the micromolar concentrations of NO_3_^−^ compared to the nanomolar concentrations of NO_2_^−^ in human skeletal muscle samples.

Samples washed with saline may provide more reliable [NO_3_^−^] and [NO_2_^−^] measurements compared to samples only blotted with gauze as contamination of NO_3_^−^ and NO_2_^−^ from blood will be reduced by the saline wash. In this study, the reliability of the [NO_3_^−^] and [NO_2_^−^] measurements were assessed by comparing the two sample processing methods with each other and to their controls (same processing method but with ddH_2_O instead of stop solution) using paired samples t−tests, Bland-Altman analysis, and correlation analysis.

The secondary aims of the study were to: a) determine the coefficient of variation (CV) between concentration measurements from different tissue samples from the same muscle (to determine the reproducibility of the measurements) and (b) examine the relationship between sample weight and [NO_3_^−^] and [NO_2_^−^] in tissue samples. Based on the observations from rodent studies mentioned previously [23], we hypothesised that there will be between-sample variability in [NO_3_^−^] and [NO_2_^−^] in different biopsies from the same human *vastus lateralis* muscle, and that the [NO_3_^−^] and [NO_2_^−^] would decrease with increasing sample weight in human skeletal muscle tissue samples.

## Methods

### Participants

Twenty-four healthy individuals (10 men and 14 women) aged 18−65 years (38 ± 16 years) volunteered to participate in this study (participants were recruitment for this study between 24^th^ Jan 2022 and 11^th^ Mar 2022). Since the aim of the study was to compare tissue processing methods from multiple biopsies obtained from the same individual, the members of the participant group were chosen to be heterogenous in terms of age, sex, physical activity, and diet. The study was approved by the Sport and Health Sciences Research Ethics Committee, University of Exeter (ref: 21-10-20-B-04), and was conducted in accordance with the principles of the Declaration of Helsinki. The experimental procedures, and related risks and benefits, were explained to all participants and written informed consent was obtained. Participants with conditions that were contraindicative for skeletal muscle biopsies (e.g., volunteers with bleeding or blood clotting disorders, or on blood thinning medication such as warfarin) were excluded from the study. Insufficient skeletal muscle tissue was obtained from the biopsies of two participants, hence skeletal muscle tissue samples from 22 participants were analysed. Due to variation in the amount of muscle tissue obtained from the participants through the biopsies, different numbers of samples were used for each analysis, and these are specified in the results.

### Preparation of stop solution

890 mM potassium ferricyanide (K_3_Fe(CN)_6_ 10330544, Thermo Scientific Acros, UK) and 118 mM N-ethylmaleimide (NEM E3876, Sigma-Aldrich, UK) were mixed in in double distilled water (ddH_2_O). A non-ionic surfactant Nonidet P40 Substitute (NP-40 15885388, Thermo Scientific Chemicals, UK) was added in a 1:9 ratio (v/v, NP-40/solution) and mixed gently to avoid foaming. This mixture was diluted in a 1:9 ratio with ddH_2_O (final concentration 89 mM potassium ferricyanide and 11.9 mM NEM) and used as the stop solution [23].

### Experimental procedures

Each participant visited the laboratory on one occasion. Two incisions were made approximately an inch apart in the m. *vastus lateralis* of each participant. Muscle tissue samples were collected by four to six biopsies from the two incisions (2–3 biopsies per incision) using the percutaneous Bergström needle technique modified for a manual vacuum [28]. The biopsy samples were processed within 10 seconds of collection and were randomized for processing order to reduce potential bias.

### Processing of muscle tissue samples

**Fig 1:**
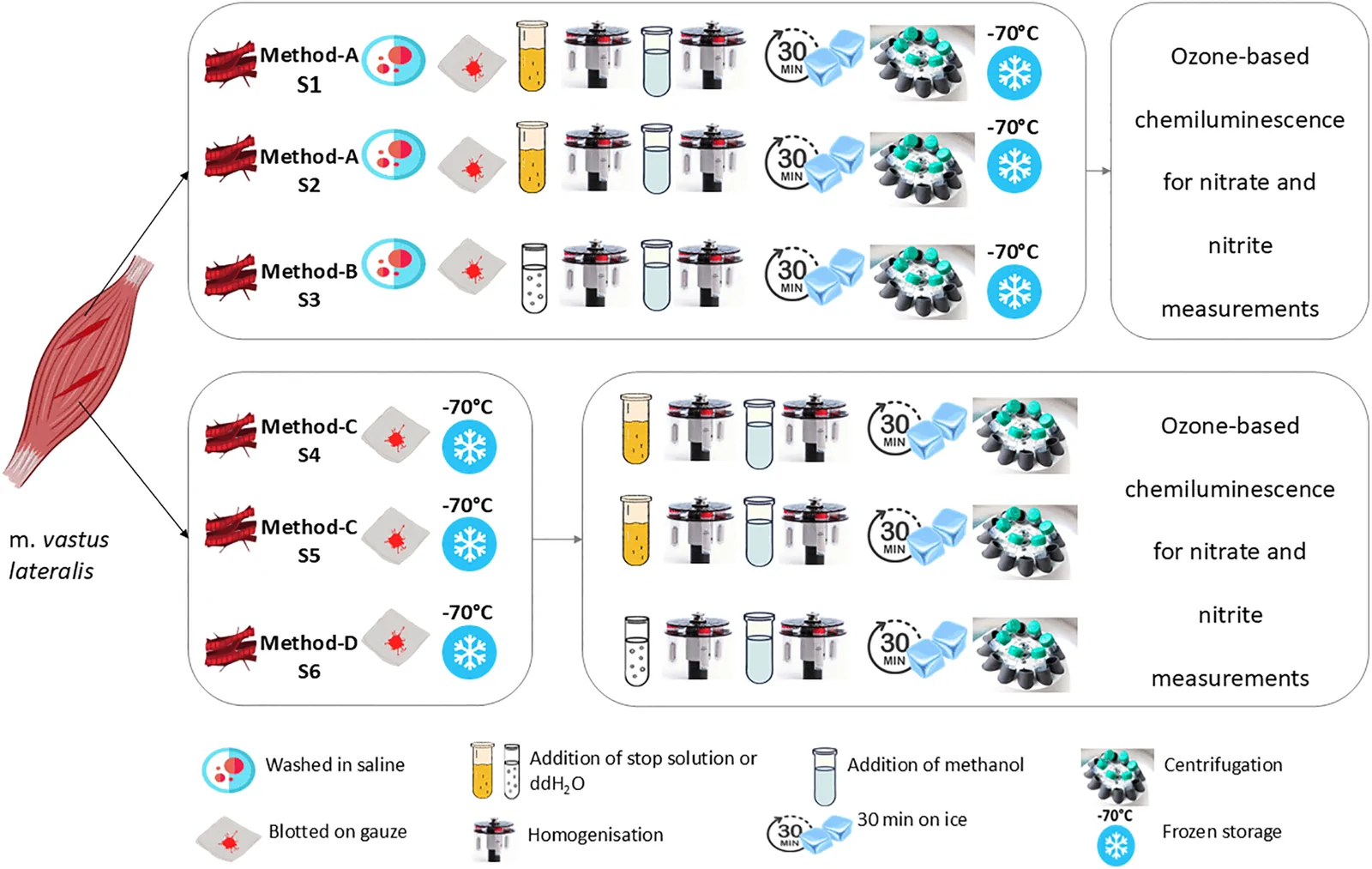
Summary of muscle tissue processing methods. Samples S1 and S2 (corresponding to Method-A) were washed in saline, blotted with sterile gauze, homogenised with stop solution and methanol, placed on ice for 30 min, centrifuged at 13,000 g at 4 °C for 30 min, and the supernatant was stored at −70 °C until the day of ozone-based chemiluminescence analysis for [NO_3_^−^] and [NO_2_^−^] measurements. Sample S3 (corresponding to Method-B) was processed in the same way as above, with stop solution replaced with ddH_2_O. Samples S4 and S5 (corresponding to Method-C), and S6 (corresponding to Method-D) were blotted with sterile gauze, snap-frozen in liquid nitrogen, and stored at −70 °C until further analysis. On the day of [NO_3_^−^] and [NO_2_^−^] measurements, samples S4 and S5 (corresponding to Method-C) were homogenised with stop solution and methanol, placed on ice for 30 min, centrifuged at 13,000 g at 4 °C for 30 min, and then the supernatant was used for ozone-based chemiluminescence analysis for [NO_3_^−^] and [NO_2_^−^] measurements. Sample S6 (corresponding to Method-D) was processed in the same way as above, with stop solution replaced with ddH_2_O.

The processing of muscle tissue samples is summarised in Fig 1. Muscle tissue samples from two separate biopsies (samples S1 and S2 processed by Method-A) and a third muscle sample (sample S3 processed by Method-B, and used as Method-A’s control) were washed in phosphate buffered saline pH 7.4 (P-3813, Sigma-Aldrich, UK) and then blotted using sterile gauze (Premier Healthcare & Hygiene Ltd, Tyne & Wear, UK) to remove blood and any visible adipose tissue. Stop solution was added to two of the tissue samples (S1 and S2 processed by Method-A) and ddH_2_O was added to the control sample (S3 processed by Method-B) in a 5:1 ratio (e.g., 250 µl stop solution or ddH_2_O was added to 50 mg wet weight muscle tissue sample) in hard tissue homogenizing tubes with 2.8 mm ceramic beads (Precellys® Lysing Kit, Bertin Technologies, Montigny-le-Bretonneux, France). The samples were then homogenised with an Analytik Jena SpeedMill Plus instrument (Analytik Jena GmbH, Germany) with a fixed g-value of 10,000 g (12,000 rpm) twice for 45 s each, briefly vortexed, and ice-cold methanol was then added in a 10:1 ratio (e.g., 500 µl methanol was added to 50 mg wet weight muscle tissue sample) for deproteinization. The samples were homogenised again for 45 s, placed on ice for 30 min, and then centrifuged (Sorvall ST16R centrifuge with a 45° fixed angle rotor of 10 cm radius) at 13,000 g at 4 °C for 30 min. The supernatant was aspirated and stored at −70 °C until the day of [NO_3_^−^] and [NO_2_^−^] measurements. The ratio of stop solution and methanol, and the homogenisation procedures are based on the protocol developed by Park *et al.*, 2021 [23] for rodent skeletal muscle.

Muscle tissue samples from two separate biopsies (samples S4 and S5 processed by Method-C) and a third muscle sample (sample S6 processed by Method-D, and used as Method-C’s control) were blotted using sterile gauze to remove blood and any visible adipose tissue. The samples were then snap-frozen in liquid nitrogen prior to being stored at −70 °C until subsequent analysis. On the day of [NO_3_^−^] and [NO_2_^−^] measurements, stop solution was added to two of the tissue samples (S4 and S5 processed by Method-C) and ddH_2_O was added to the control sample (S6 processed by Method-D) in a 5:1 ratio (e.g., 250 µl stop solution or ddH_2_O was added to 50 mg wet weight muscle tissue sample) in hard tissue homogenizing tubes with 2.8 mm ceramic beads. The samples were then homogenised twice for 45 s each, briefly vortexed, and ice-cold methanol was then added in a 10:1 ratio (e.g., 500 µl methanol was added to 50 mg wet weight muscle tissue sample) for deproteinization. The samples were homogenised again for 45 s, placed on ice for 30 min, and then centrifuged at 13,000 g at 4 °C for 30 min. The supernatant was aspirated and used for [NO_3_^−^] and [NO_2_^−^] measurements.

### [NO_3_^−^] and [NO_2_^−^] determination

A Sievers gas-phase chemiluminescence NO analyser (Sievers 280i Nitric Oxide Analyser, GE Analytical Instruments, Boulder, CO, USA) was used for ozone-based chemiluminescence quantification of [NO_3_^−^] and [NO_2_^−^] as per previously described methods [14,29,30]. The aspirated supernatant from the muscle tissue samples were injected through a purge vessel containing the reducing agent vanadium chloride for [NO_3_^−^] or tri-iodide solution for [NO_2_^−^] measurements. Samples from each method were processed sequentially due to differences in the sample preparation protocols. To negate any effects of stop solution and ddH_2_O on [NO_3_^−^] and [NO_2_^−^] measured in the samples, [NO_3_^−^] and [NO_2_^−^] were measured in the stop solution and ddH_2_O used and deducted from the sample concentrations. Data analysis for concentrations calculations was performed at the end of all chemiluminescence measurements.

### Statistical analysis

Paired samples t-tests were used to determine differences between concentrations measured using Method-A (mean of samples S1 and S2) and its control, Method-B (S3); Method-C (mean of samples S4 and S5) and its control, Method-D (S6); and Method-A (mean of samples S1 and S2) and Method-C (mean of samples S4 and S5) for both NO_3_^−^ and NO_2_^−^. Paired samples t-tests were also used to determine differences between CV from concentrations measured using Method-A (mean of samples S1 and S2) and Method-C (mean of samples S4 and S5) for both NO_3_^−^ and NO_2_^−^.

A Bland-Altman analysis was used to assess the agreement between the concentrations measured using Method-A (mean of samples S1 and S2) and its control, Method-B (S3); Method-C (mean of samples S4 and S5) and its control, Method-D (S6); and Method-A (mean of samples S1 and S2) and Method-C (mean of samples S4 and S5) for both NO_3_^−^ and NO_2_^−^. A regression-based Bland-Altman was used to check for proportional bias. According to Bland and Altman [31], good agreement between the methods is accepted when the mean of the difference (bias) is close to zero and 95% of the individual differences are within the limits of agreement (LOA). In our study, a bias value close to zero was defined as ± 1.5 nmol/g to represent a minimal systematic bias between measurements.

The Pearson product moment correlation coefficient was calculated to assess relationships between concentrations measured using Method-A (mean of samples S1 and S2) and its control, Method-B (S3); Method-C (mean of samples S4 and S5) and its control, Method-D (S6); and Method-A (mean of samples S1 and S2) and Method-C (mean of samples S4 and S5) for both NO_3_^−^ and NO_2_^−^. The Pearson product moment correlation coefficient was also calculated to assess relationships between the sample weight and concentrations measured using Method-A and Method-C for both NO_3_^−^ and NO_2_^−^. Data normality was assessed by the Shapiro-Wilk test. If data were not normally distributed, Spearman’s correlation coefficient was calculated.

Within-participant reliability was assessed using intra-participant CV (variation in measurement between two samples from the same person, measured using the same method) and compared to analytical CV (variation between two measurements of the same sample, measured using the same method). Coefficient of repeatability (CR, calculated as 1.96 * √2 * SD) and the intra-class correlation coefficient (ICC) were also used to support within-participant reliability.

GraphPad Prism (GraphPad Software Version 9 for Windows, Boston, Massachusetts, USA) was used for statistical analysis of data. The alpha level to denote statistical significance was set at p < 0.05. All results are expressed as mean  ±  standard deviation (SD).

## Results

### [NO_3_^-^] in muscle tissue samples

**Fig 2:**
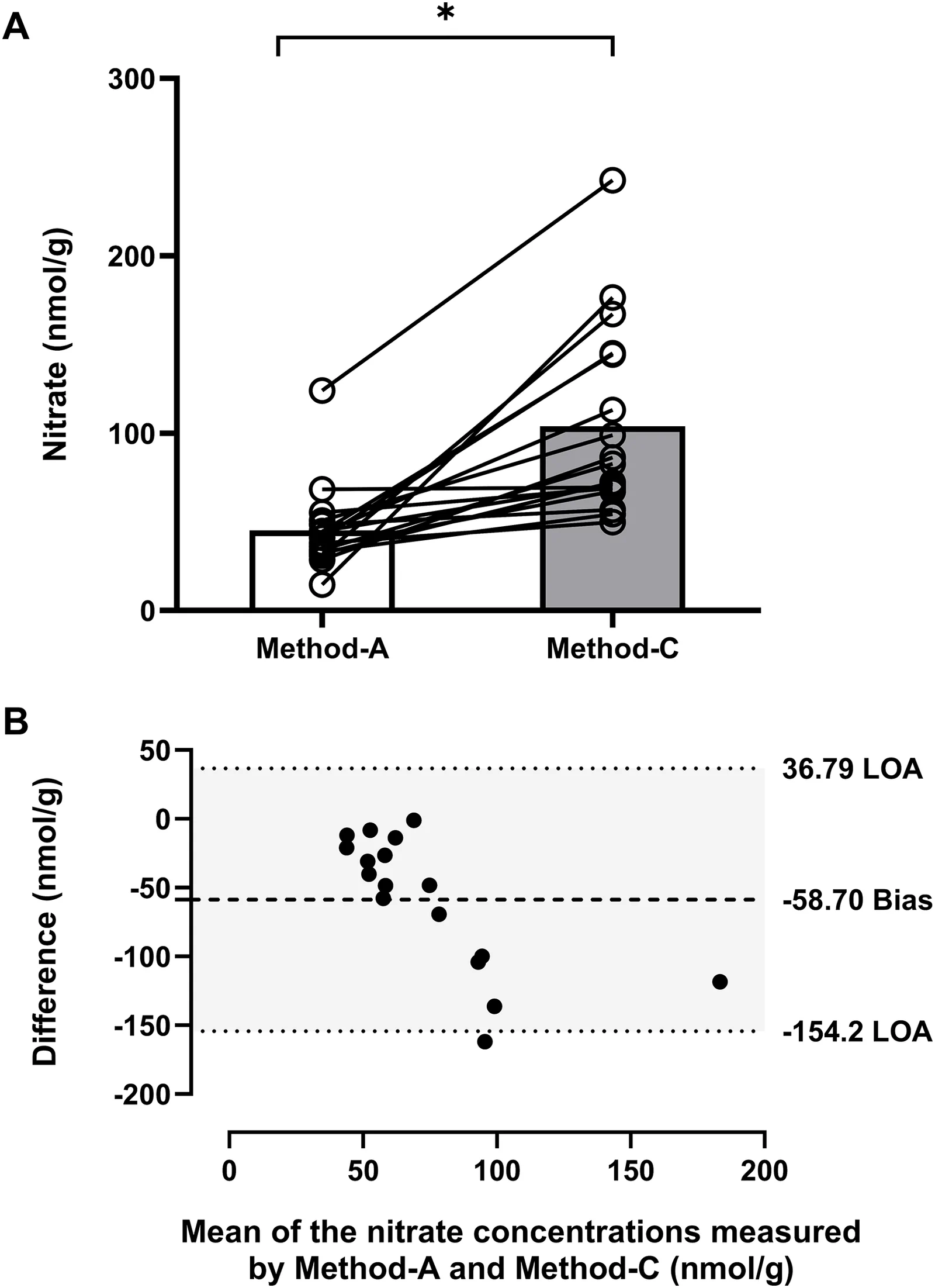
Comparison of the [NO_3_^−^] in muscle tissue lysates after two different methods of sample processing. **Panel A:** [NO_3_^−^] measured after tissue processing by Method-A vs Method-C. The bars represent the mean concentrations, and the circles represent the individual sample concentrations. **Panel B:** Bland-Altman comparison between the [NO_3_^−^] measured after tissue processing by Method-A vs Method-C. LOA: 95% Limits of agreement. *represents a statistically significant difference between the means (p < 0.01). n = 17 participants for Methods A and C, with two samples per participant processed by each method.

The mean [NO_3_^−^] in muscle tissue samples was significantly lower when measured by Method-A (45 ± 23 nmol/g) compared to Method-C (104 ± 52 nmol/g; *p < 0.01*; Fig 2A) (n = 17 participants for Methods A and C, with two samples per participant processed by each method). There was no significant correlation (*r*_*s*_ *= −0.09 [95% CI: −0.56, 0.42], p = 0.72*) and a high systematic and proportional bias with a relatively wide range for the LOA (Bias: −58.70 ± 48.72 nmol/g, LOA: 36.79, −154.20 nmol/g; Fig 2B) between the [NO_3_^-^] measured by the two methods. The limit of detection and limit of quantification for [NO_3_^−^] based on calibrations using standard concentrations of reagent grade NaNO_3_ are presented in the supplementary information S1 File.

### Effect of stop solution on the [NO_3_^-^] in muscle tissue samples

**Fig 3:**
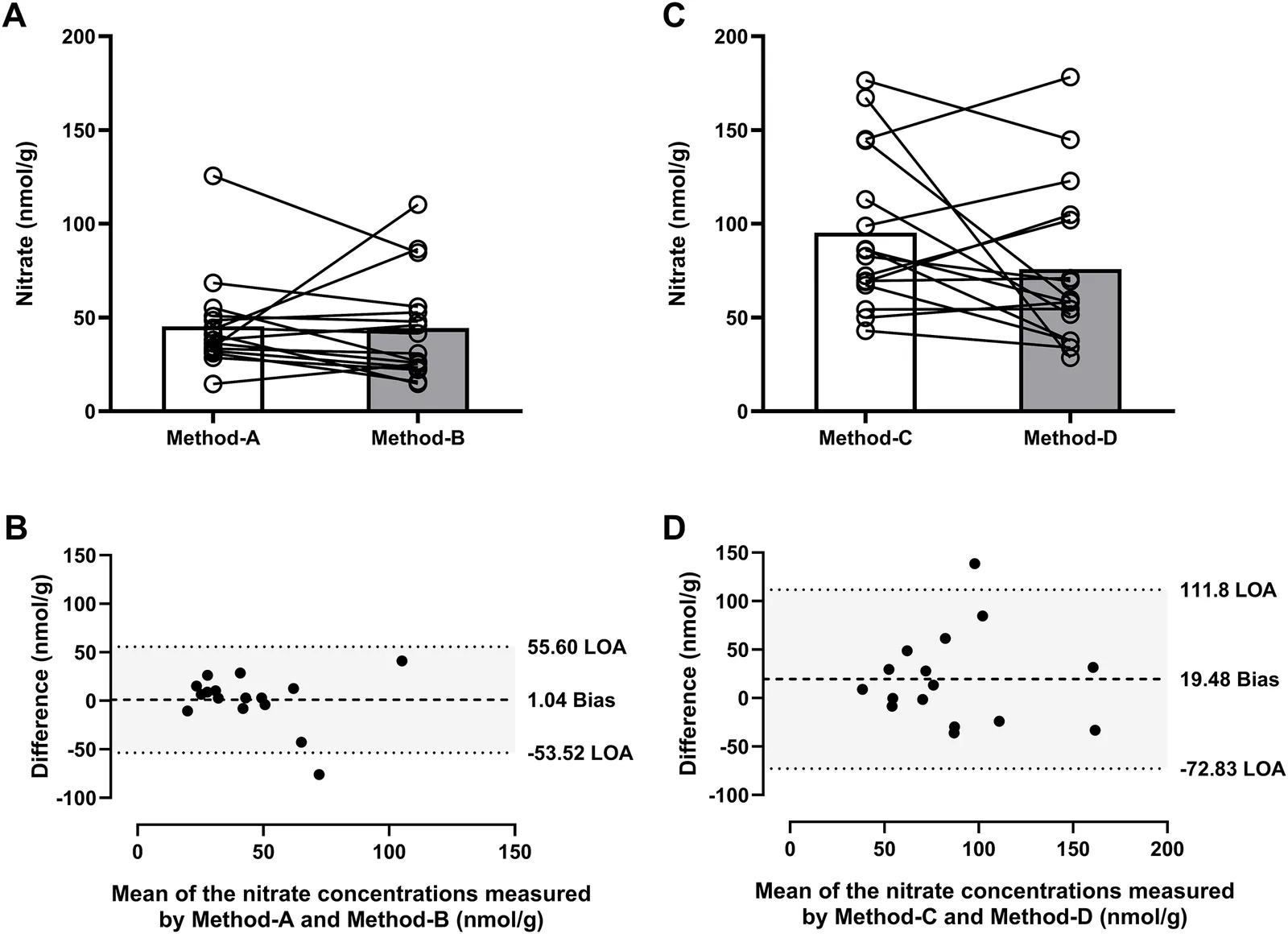
Effect of stop solution on [NO_3_^−^] in muscle tissue lysates. **Panel A:** [NO_3_^−^] measured after tissue processing by Method-A vs Method-B. The bars represent the mean concentrations, and the circles represent the individual sample concentrations. **Panel B:** Bland-Altman comparison between the [NO_3_^−^] measured after tissue processing by Method-A vs Method-B. **Panel C:** [NO_3_^−^] measured after tissue processing by Method-C vs Method-D. The bars represent the mean concentrations, and the circles represent the individual sample concentrations. **Panel D:** Bland-Altman comparison between the [NO_3_^−^] measured after tissue processing by Method-C vs Method-D. LOA: 95% Limits of agreement. n = 16 participants for Method-A, Method-B, Method-C, and Method-D, with two samples per participant processed by Methods A and C, and one sample per participant processed by Methods C and D.

The addition of stop solution made no significant difference to the [NO_3_^-^], compared to the control, when measured by Method-A (Fig 3A) or Method-C (Fig 3C) (n = 16 participants for Methods A, C, and their controls, with two samples per participant processed by Methods A and C, and one sample per participant processed for controls). There was a high inter-sample variability between [NO_3_^-^] measured with and without stop solution (Fig 3A and 3C). Method-A correlated with Method-B (*r*_*s*_ *= 0.58 [95% CI: 0.10, 0.84], p = 0.02*) with a minimal systematic bias and a relatively narrower range for the LOA (Bias: 1.04 ± 27.84 nmol/g, LOA: 55.60, −53.52 nmol/g; Fig 3B) between the [NO_3_^-^] measured with and without stop solution. In contrast, Method-C did not correlate with Method-D (*r*_*s*_ *= 0.29 [95%CI: −0.26, 0.69], p = 0.28*) with a higher systematic bias and a relatively wider range for the LOA (Bias: 19.48 ± 47.10 nmol/g, LOA: 111.80, −72.83 nmol/g; Fig 3D) between the [NO_3_^-^] measured with and without stop solution.

### [NO_2_^-^] in muscle tissue samples

**Fig 4:**
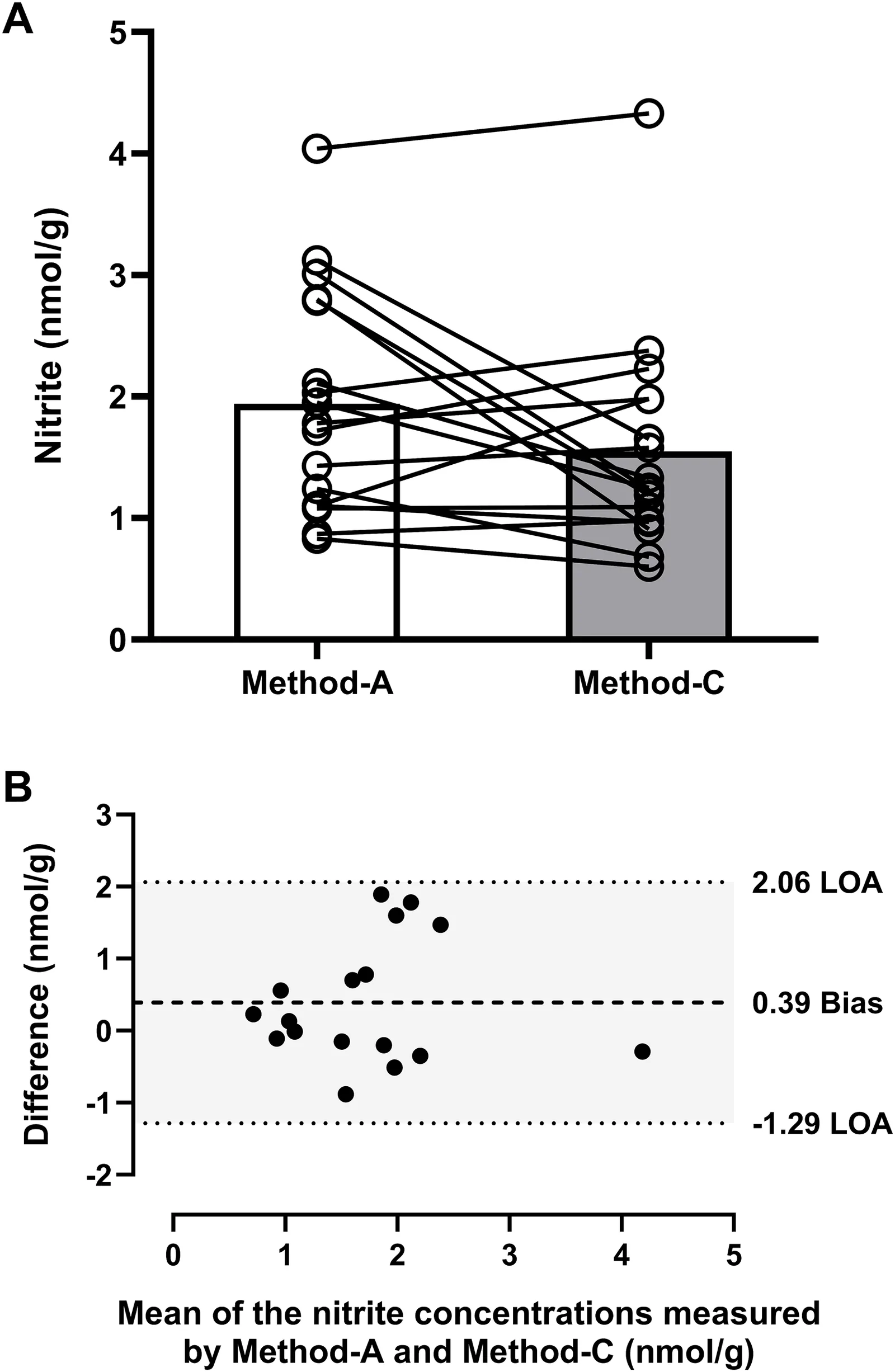
Comparison of the [NO_2_^−^] determined in muscle tissue lysates after two different methods of sample processing. **Panel A:** [NO_2_^−^] measured after tissue processing by Method-A vs Method-C. The bars represent the mean concentrations, and the circles represent the individual sample concentrations. **Panel B:** Bland-Altman comparison between the [NO_2_^−^] measured after tissue processing by Method-A vs Method-C. LOA: 95% Limits of agreement. n = 17 participants for Methods A and C, with two samples per participant processed by each method.

The mean [NO_2_^-^] values in muscle tissue samples were not significantly different when measured by Method-A (1.94 ± 0.90 nmol/g) compared to Method-C (1.55 ± 0.86 nmol/g; Fig 4A) (n = 17 for Methods A and C, with two samples per participant processed by each method). The range of [NO_2_^-^] was wider when measured by Method-A compared to Method-C. Four of the five highest [NO_2_^-^] values obtained by Method-A gave lower concentrations when measured by Method-C (Fig 4A). There was no significant correlation (*r*_*s*_ *= 0.45 [95% CI: −0.06, 0.77], p = 0.99*) and minimal systematic bias with a relatively wide range for the LOA (Bias: 0.39 ± 0.85 nmol/g, LOA: 2.06, −1.29 nmol/g; Fig 4B) between the [NO_2_^-^] measured by the two methods. The limit of detection and limit of quantification for [NO_2_^−^] based on calibrations using standard concentrations of reagent grade NaNO_2_ are presented in the supplementary information S1 File.

### Effect of stop solution on the [NO_2_^-^] in muscle tissue samples

**Fig 5:**
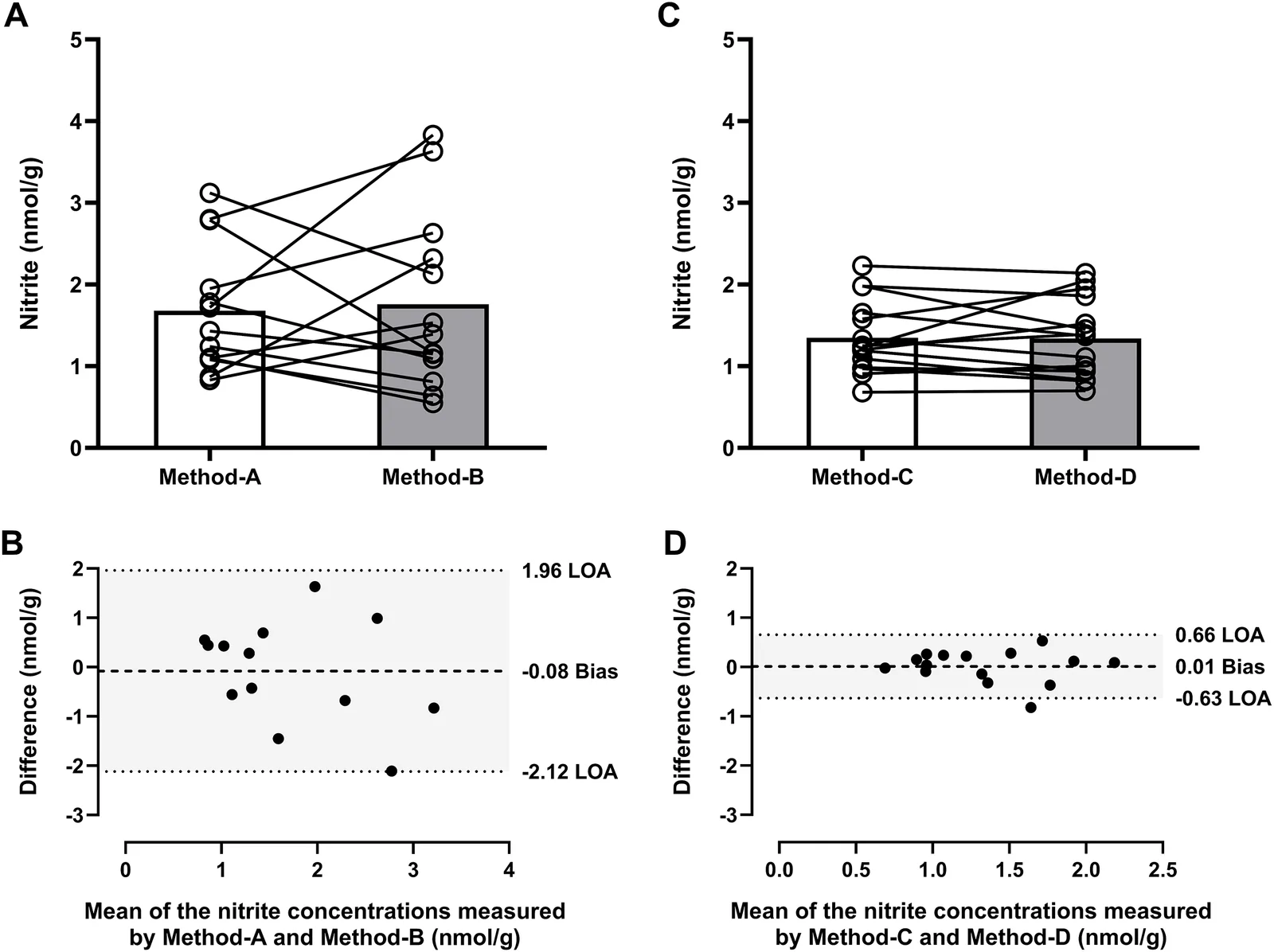
Effect of stop solution on [NO_2_^−^] in muscle tissue lysates. **Panel A:** [NO_2_^−^] measured after tissue processing by Method-A vs Method-B. The bars represent the mean concentrations, and the circles represent the individual sample concentrations. **Panel B:** Bland-Altman comparison between the [NO_2_^−^] measured after tissue processing by Method-A vs Method-B. **Panel C:** [NO_2_^−^] measured after tissue processing by Method-C vs Method-D. The bars represent the mean concentrations, and the circles represent the individual sample concentrations. **Panel D:** Bland-Altman comparison between the [NO_2_^−^] measured after tissue processing by Method-C vs Method-D. LOA: 95% Limits of agreement. n = 13 for Method-A and Method-B, n = 15 for Method-C and Method-D, with two samples per participant processed by Methods A and C, and one sample per participant processed by Methods B and D.

The addition of stop solution made no significant difference to the [NO_2_^-^], compared to control, when measured by Method-A (Fig 5A) or Method-C (Fig 5C) (n = 13 participants for Method A and its control, n = 15 participants for Method C and its control, with two samples per participant processed by Methods A and C, and one sample per participant processed for controls). There was a high inter-sample variability between [NO_2_^−^] measured with and without stop solution (Fig 5A and 5C). [NO_2_^-^] measured with and without stop solution showed no statistically significant correlation when measured by Method-A (*r*_*s*_ *= 0.37 [95%CI = −0.25, 0.77], p = 0.22*), while they showed a significant correlation when measured by Method-C (*r*_*s*_ *= 0.79 [95%CI: 0.45 to 0.93], p < 0.01*). There was minimal systematic bias between the [NO_2_^-^] measured with and without stop solution using Method-A (Bias: −0.08 ± 1.04 nmol/g) and Method-C (Bias: 0.01 ± 0.33 nmol/g; Fig 5B and 5D), while the LOA was 3.2x narrower with Method-C (LOA: 0.66, −0.63 nmol/g) compared to Method-A (1.96, −2.12 nmol/g).

### Coefficient of variation of concentration measurements from two distinct muscle tissue samples

The mean CV for [NO_3_^-^] and [NO_2_^-^] measured in different muscle samples from the same participant were not significantly different when measured by Method-A ([NO_3_^-^]: 25 ± 17% and [NO_2_^-^]: 23 ± 18%) and Method-C ([NO_3_^-^] 27 ± 19% and [NO_2_^-^] 12 ± 8%), albeit, these CVs are considerably higher than the analytical CV of 2–3% (variation between two repeated measurements of the same sample). The CR and ICC data to support within-participant reliability are presented in the supplementary information S1 File.

### Effect of sample weight on [NO_3_^-^] and [NO_2_^-^]

Both [NO_3_^-^] and [NO_2_^-^] were negatively correlated with sample weight when measured by Method-A ([NO_3_^−^]: *r*_*s*_ *= −0.53 [95%CI: −0.74, −0.25], p < 0.01* and [NO_2_^−^]: *r*_*s*_ *= −0.33 [95%CI: −0.60, −0.001], p = 0.04*), but no significant correlation was observed when measured by Method−C ([NO_3_^−^]: *r*_*s*_ *= −0.19 [95%CI: −0.48, 0.14], p = 0.25* and [NO_2_^−^]: *r*_*s*_ *= −0.22 [95%CI: −0.51, 0.11], p = 0.17*).

## Discussion

The aims of this study were to: (a) analyse the effect of saline wash and immediate addition of stop solution on skeletal muscle [NO_3_^−^] and [NO_2_^−^] measurements, (b) evaluate the reliability and reproducibility of these measurements, and (c) assess possible relationships between [NO_3_^−^] and [NO_2_^−^] and sample weight. We found that washing the human skeletal muscle tissue with saline before blotting with sterile gauze, in combination with the addition of stop solution and homogenisation immediately on sample collection, resulted in lower [NO_3_^−^] (but not higher [NO_2_^−^]) compared to samples only blotted with gauze. Addition of stop solution made no significant difference to the [NO_3_^−^] and [NO_2_^−^], compared to a control where ddH_2_O was added instead of the stop solution. Saline wash and immediate addition of the stop solution did not change the reproducibility of [NO_3_^−^] and [NO_2_^−^] measurements, compared to samples only blotted with gauze, but resulted in negative correlations between [NO_3_^−^] and sample weight, and [NO_2_^−^] and sample weight. We did not assess markers of blood contamination, and our findings would therefore benefit from verification by measurement of Hb and Mb in samples processed by the two methods.

### Saline wash and the timing of the addition of stop solution when processing skeletal muscle samples for [NO_3_^−^] and [NO_2_^−^] measurements

Saline wash followed by blotting with gauze and immediate homogenisation with stop solution resulted in significantly lower [NO_3_^−^], compared to samples only blotted with gauze and homogenised with stop solution on the day of [NO_3_^−^] and [NO_2_^−^] measurements. This suggests that washing with saline, followed by blotting with sterile gauze, is more effective in removing blood (and hence Hb) than blotting alone. This possibility requires confirmation in future studies by measuring the Hb concentration in the samples processed by the two methods. The earlier addition of stop solution may be more effective in preventing the oxidation of NO_2_^−^ to NO_3_^−^. The decrease in [NO_3_^−^] with saline wash could also be due to the removal of blood and other extracellular tissue containing NO_3_^−^ from the tissue samples, and not due to the prevention of oxidation of NO_2_^−^ to NO_3_^−^. The lack of correlation and the high systematic and proportional bias with a relatively wide range for the LOA (117.4 nmol/g) between the NO_3_^−^ concentrations measured by the two methods supports the hypothesis that saline wash and earlier addition of stop solution improves the reliability of [NO_3_^−^] measurements in human skeletal muscle tissue samples.

A saline wash and immediate homogenisation with the stop solution had no significant effect on the [NO_2_^−^], compared to samples only blotted with gauze and subsequently homogenised with the stop solution on the day of [NO_3_^−^] and [NO_2_^−^] measurements. Although a saline wash resulting in a decrease in [NO_3_^−^] seems contradictory to no change in [NO_2_^−^], these results could have possible explanations. As mentioned previously, the decrease in [NO_3_^−^] with saline wash could be due to the removal of extracellular NO_3_^−^ from the tissue samples, leading to no changes in [NO_2_^−^]. Alternatively, prevention of NO_2_^−^ oxidation to NO_3_^−^ could potentially increase [NO_2_^−^], while removal of blood containing NO_2_^−^ from the skeletal muscle samples could potentially decrease [NO_2_^−^]. These counteracting factors could neutralize one another resulting in no overall changes in [NO_2_^−^]. It is also possible that [NO_2_^−^] measurements are sensitive to the kinetic limitations of K_3_Fe(CN)_6_ penetration, efficiency of sample deproteinization, and the role of freezing duration on NO_2_^−^ stability, however samples in this study were frozen for a similar period of time and hence storage time is unlikely to have affected our results.

The [NO_2_^−^] from samples measured by the two methods showed limited bias in the Bland−Altman analysis, but there was no correlation between the [NO_2_^−^] measured by the two methods. There was a wide range of [NO_2_^−^] in samples washed in saline compared to samples only blotted with sterile gauze. The samples with higher [NO_2_^−^] after saline wash had lower concentrations when only blotted with sterile gauze. These indicate that in samples with higher [NO_2_^−^], the earlier addition of the stop solution could be preventing NO_2_^−^ oxidation, thereby preserving NO_2_^−^. In conclusion, the preservation of NO_2_^−^ in samples with a higher [NO_2_^−^] suggests Method−A is preferred for the assessment of [NO_2_^−^] in human skeletal muscle samples.

In some studies that measured [NO_3_^−^] and [NO_2_^−^] in human skeletal muscle samples, biopsied skeletal muscle was snap frozen and homogenised without stop solution before concentration measurements [32–34]. Nyakayiru *et al.* [11] freeze−dried the samples, removed blood and other tissue under a dissecting microscope and homogenised the samples with perchloric acid before making [NO_3_^−^] and [NO_2_^−^] measurements, without the use of stop solution. Wylie *et al.* [12] blotted the muscle biopsy samples using sterile gauze, snap−froze the samples in liquid nitrogen, and homogenised the samples in a mixture of concentrated stop solution and methanol before chemiluminescence analysis. In other studies [13,14,24–27], muscle biopsy samples were blotted using sterile gauze, snap-frozen in liquid nitrogen, and homogenised in a mixture of diluted stop solution and methanol on the day of the chemiluminescence analysis following the homogenisation procedures developed by Park et al. [23]. This latter procedure is similar to Method-C in the present study. In comparison to these procedures, our results indicate that a saline wash before blotting the samples with sterile gauze in conjunction with an earlier addition of the diluted stop solution and homogenisation before storage, may improve the reliability of [NO_3_^−^] and [NO_2_^−^] measurements in human skeletal muscle tissue samples. These procedures may aid in removing more haemoproteins from human tissues. In rodents, this is achieved by perfusing of rodent organs with saline before tissue extraction [9,10,15,23]. Since this obviously cannot be performed in human tissues, saline wash and immediate homogenisation with stop solution provides an alternative method to removing the effect of haemoproteins in human tissues.

### Use of stop solution when processing skeletal muscle samples for [NO_3_^−^] and [NO_2_^−^] measurements

There was no significant difference in skeletal muscle [NO_3_^−^] or [NO_2_^−^] measured with or without the stop solution. However, the inter-sample variability suggests the addition of the stop solution could still be an important factor in a few individual samples.

For [NO_3_^−^] measurements, when the samples were washed with saline and immediately homogenised with stop solution, the smaller inter-sample variability (Fig 3A), minimal bias with a relatively narrower range for the LOA (109.1 nmol/g) (Fig 3B) compared to Method-C and its control (184.6 nmol/g), and a significant correlation between the [NO_3_^−^] measured with and without the stop solution, indicates the results from these methods were well aligned. Hence, the presence of stop solution may be considered less critical when the samples are washed with saline, blotted, and immediately homogenised. However, when the samples were only blotted with sterile gauze, the higher inter-sample variability (Fig 3C), the higher bias with a relatively wider range for the LOA (184.6 nmol/g) (Fig 3D), and the lack of a correlation between [NO_3_^−^] measured with and without the stop solution suggests that there may be a subset of samples in which unknown factor(s) entail a beneficial effect of the stop solution. This needs further investigation.

For [NO_2_^−^] measurements, when the samples were washed with saline and immediately homogenised with stop solution, the higher inter-sample variability (Fig 5A) and the lack of correlation between [NO_2_^−^] measured with and without the stop solution, suggests that the stop solution could be more beneficial when samples are washed with saline and immediately homogenised with stop solution. When the samples were only blotted with sterile gauze, the smaller inter-sample variability (Fig 5C) and the significant correlation between the [NO_2_^−^] measured with and without the stop solution indicates the results from these methods were well aligned. Hence the stop solution could be considered less critical when the samples are only blotted with sterile gauze.

Stop solution has been used during homogenisation in many studies measuring [NO_3_^−^] or [NO_2_^−^] in human skeletal muscle since 2019 [12–14,24–26]. In our study, there is no indication of what might cause the stop solution to be more important for obtaining more accurate [NO_3_^−^] and [NO_2_^−^] in some samples compared to others, but the addition of the stop solution had no adverse effects on [NO_3_^−^] or [NO_2_^−^] measurements in the other samples. Hence it is prudent to process all samples with stop solution for [NO_3_^−^] and [NO_2_^−^] measurements.

### Reproducibility of [NO_3_^−^] and [NO_2_^−^] measurements

There was no difference in reproducibility assessed by the CV of [NO_3_^−^] and [NO_2_^−^] measurements between Method−A and Method-C. However, the ~ 20% CV for concentrations measured in samples from the same participant (intra-individual CV) compared to the analytical CV of 2–3% (inter-assay CV) could be due to variability in immediate processing of biopsied samples in terms of the amount of blood removed by saline wash and blotting. This could be analysed in future studies by measuring the Hb content in processed tissue samples. Another cause for the increased CV could be the variability in the [NO_3_^−^] and [NO_2_^−^] within the same muscle group, possibly due to differences in fibre type in the tissue obtained from the different biopsies [35]. The CV from either of these methods is appreciably lower than increases in concentrations measured post-NO_3_^−^ supplementation in previous studies [11–14,24–27].

### Effect of sample weight on [NO_3_^−^] and [NO_2_^−^] measurements

For every 1 mg increase in sample weight, the mean [NO_3_^−^] in skeletal muscle samples showed a decrease of 0.96 ± 0.39 nmol/g and the mean [NO_2_^−^] concentration showed a decrease of 0.01 ± 0.01 nmol/g. The decrease in [NO_3_^−^] and [NO_2_^−^] with increasing sample weight could be due to differences in the efficiency of lysing of cells during sample homogenisation, non−uniform fibre type distribution within larger tissue samples, variation in extracellular space across samples, or diffusion limitations for the stop solution or methanol. In future studies, the sample weight should be kept consistent between samples to increase the reliability of the [NO_3_^−^] and [NO_2_^−^] measurements. This can be done by processing a similar amount of tissue irrespective of biopsy sample weight by combining samples below the threshold weight or splitting the biopsy samples into threshold weight samples, before processing them for [NO_3_^−^] and [NO_2_^−^] measurements.

### Experimental considerations

The focus of the present study was on reliable assessment of human skeletal muscle [NO_3_^−^] and [NO_2_^−^] measurements by comparing two different methods to each other and to each of their respective control methods, to determine protocol robustness. We assessed reproducibility by comparing the CV from the two different methods to measure test-retest variability. However, we did not assess validity of the [NO_3_^−^] and [NO_2_^−^] measurements (as there is no established reference method) such that the primary outcomes of this study constitute a methodological advance only. Establishing technical reliability of a method must precede assessment of validity. Future research is required to validate the sensitivity of this method to detect meaningful changes in muscle [NO_3_^−^] and [NO_2_^−^], e.g., between different participant cohorts or following dietary supplementation. [NO_3_^−^] and [NO_2_^−^] in blood, interstitial fluid, and tissues other than skeletal muscle that are present in an unprocessed biopsy sample also have biological relevance, but providing insight into physiological relevance of NO_3_^−^ and NO_2_^−^ in those compartments was beyond the scope of this study. We believe that developing a reliable technique for isolating the skeletal muscle compartment will enable future studies to identify potentially meaningful relationships with muscle function with greater accuracy.

The methodologies used in this study address some of the variations in [NO_3_^−^] and [NO_2_^−^] that are a result of tissue processing methods. Variations could also arise from tissue homogenisation methods, homogenisation buffers, and measurement methods, and some of these considerations have been investigated previously [23,36]. We did not use HPLC as an orthogonal validation method as the ferricyanide (in the stop solution) is incompatible with the methods that use post−column cadmium-mediated NO_3_^−^ reduction to NO_2_^−^ [37]. All samples in this study were homogenised with a bead homogeniser and concentrations measured using ozone-based chemiluminescence. Hence the variations in the measurements in the current study were reflective of the two methods of tissue processing compared in this study.

## Conclusion

Saline washing of biopsied tissue samples improved the reliability of nitrate and nitrite concentrations measured in human skeletal muscle. It is therefore recommended that human muscle tissue samples collected for [NO_3_^−^] and [NO_2_^−^] measurements be washed with saline and immediately homogenised with stop solution before storage. It is acknowledged that the findings of this study would benefit from verification by measurement of Hb and Mb in samples processed by the two methods. The amount of saline required and time period of the wash to remove blood contamination in the samples may also need refinement for establishing consistency between samples. The extraction efficiency of the homogenisation procedures used in this study should be compared with samples of various weights to refine the procedure for maximum extraction in higher weight samples. Until such procedures are developed, the sample weight should be kept consistent between the samples, to increase the reliability of [NO_3_^−^] and [NO_2_^−^] measurements.

## Supporting information

S1 File Supplementary information containing [NO_3_^−^], [NO_2_^−^] and wet weight for individual samples, calibration curves and LOD/LOQ calculations [NO_3_^−^] and [NO_2_^−^] for standards, and calculations for analytical CV, Coefficient of repeatability (CR) and the Intra-class correlation coefficient (ICC). (XLSX)
